# IMP3 promotes re‐endothelialization after arterial injury via increasing stability of VEGF mRNAhv

**DOI:** 10.1111/jcmm.17225

**Published:** 2022-03-22

**Authors:** Xinmiao Zhou, Qingqing Ye, Jinlei Zheng, Lin Kuang, Jianhua Zhu, Hui Yan

**Affiliations:** ^1^ Department of Cardiology The First Affiliated Hospital College of Medicine Zhejiang University Hangzhou China; ^2^ Department of Intensive Care Unit The First Affiliated Hospital College of Medicine Zhejiang University Hangzhou China

**Keywords:** endothelial cells, IMP3, mRNA stability, post‐angioplasty restenosis, re‐endothelialization

## Abstract

IMP3, an RNA‐binding protein (RBP) that participates in the process of post‐transcriptional modifications of mRNA transcripts, is capable of altering cellular functions, and in some cases, be involved in specific disease progression. We aimed to investigate whether IMP3 has the ability to regulate the functional properties of endothelial cells and re‐endothelialization in response to arterial injury. Wire injury was introduced to the right carotid arteries of wildtype C57/BL6 mice. As a result, IMPs’ expressions were up‐regulated in the induced arterial lesions, and IMP3 was the most up‐regulated RNA among other IMPs. We overexpressed IMP3 before the wire‐injured surgery using adeno‐associated virus AAV2‐IMP3. In vivo studies confirmed that IMP3 overexpression accelerated the progress of re‐endothelialization after arterial injury. In vitro, endothelial cells were transfected with either ad‐IMP3 or Si‐IMP3, cell functional studies showed that IMP3 could promote endothelial cell proliferation and migration, while reducing apoptosis. Mechanistic studies also revealed that IMP3 could enhance VEGF mRNA stability and therefore up‐regulate activities of VEGF/PI3K/Akt signalling pathway. Our data indicated that IMP3 promotes re‐endothelialization after arterial injury and regulates endothelial cell proliferation, migration and apoptosis via increasing stability of VEGF mRNA and activation of VEGF/PI3K/Akt signalling pathway.

## 1 INTRODUCTION

Coronary angioplasty and stents implantation are widely used surgical techniques for restoring blood flow to the coronary arteries; it is done by mechanically widening the narrowed or blocked segments of the artery to alleviate myocardial ischemia. Although the use of bare metal stents (BMS) in most procedures of myocardial revascularization were effective in reversing acute vessel closures, whilst lowering risks of coronary complications, at the same time, stenting procedures were frequently plagued with neointimal hyperplasia and in‐stent restenosis^1^ post‐surgery, as a result of excessive vascular healing in response to stent‐related injuries. More recently, drug‐eluting stents (DES) were introduced as a safer, more effective alternative to BMS, with statistically superior performance and significantly reduced risk of restenosis.^2^ Drug‐eluting stents are often coated with cytotoxic or cytostatic drugs, aiming to prevent vascular smooth muscle cells (VSMC) proliferation and neointimal hyperplasia thereby decreasing the risk of in‐stent restenosis. However, due to the non‐selective nature of these drugs, DES also impairs the normal healing response, resulting in increased risks of late‐ and very late‐onset stent thrombosis.^3^, ^4^, ^5^ Unfortunately, striving a balance between vascular healing response and thrombosis had proven to be a difficult challenge and an urgent problem that needs to be resolved.

Insulin‐like growth factor 2 mRNA‐binding proteins (IMPs, including IMP1, IMP2 and IMP3) are a family of RNA‐binding proteins (RBPs) that participate in post‐translational RNA processing, such as transcript localization, translation and stabilization.^6^, ^7^ IMPs, composed of six canonical RNA‐binding domains, including two RNA recognition motif (RRM) domains and four K homology (KH) domains, are a highly conserved family of single‐stranded RNA‐binding proteins.^7^, ^8^ Previous research have revealed that IMPs carry numerous roles in cellular metabolism,^9^, ^10^ adhesion, survival,^11^ maintenance,^12^ differentiation^13^ and carcinogenesis.^7^, ^14^ Over the years, IMPs have become increasingly relevant in the field of cardiovascular disease research. For instance, Meng Li et al demonstrated that IMP2 contributed to DOX‐induced cardiotoxicity upon completion of chemotherapy, while Hosen et al revealed that IMP2 and Airn (a long non‐coding RNA) co‐regulate physiological functions of cardiomyocytes^15^; furthermore, Wang and colleagues identified IMP3 as a previously unrecognized regulator of cardiomyocyte proliferation.^16^ Additionally, IMP1 is identified as a potent up‐regulator of macrophage motility. Given the vast physiological significance and emerging evidence of RBPs being identified in key regulatory pathways in cardiovascular health, as well as cardiovascular diseases, it is pivotal for this repertoire of knowledge to be deepened, in hope of discovering novel therapeutic targets. To this date, the expression profiles of IMPs family members during the process of re‐endothelialization and their roles in endothelial cell functions had remained unclarified.^17^ Therefore, we aim to compare expression levels of several IMPs transcripts in wire‐injured lesions, as well as to investigate their physiological functions and to investigate the underlying mechanisms of how IMPs could influence the process of re‐endothelialization. Here, our study demonstrated that by up‐regulating IMP3 expression, re‐endothelialization was enhanced in vivo whilst enhanced endothelial cell proliferation and migration were observed in vitro; furthermore, additional mechanistic studies also revealed the role of IMP3 in promoting VEGF mRNA stabilization.

## 2 MATERIALS AND METHODS

### 2.1 Animals

All animal experiments were approved by the Animal Care Ethics Committee of the First Affiliated Hospital, College of Medicine, Zhejiang University, China. Wild type C57BL/6 mice were purchased from Shanghai Laboratory Animal Research Center (Shanghai, China), and housed in the First Affiliated Hospital, College of Medicine, Zhejiang University. All mice were maintained under s temperature, humidity, 12:12‐h dark‐light cycle, and were provided with an appropriate amount of water and mouse diet.

Mice were injected with AAV2‐IMP3 or AAV2‐Control seven days before surgery, each injection contains 5 × 10^11^ viral particles diluted in 100 μL of sterile phosphate‐buffered saline (PBS).

### 2.2 Endothelial denudation and measurement of neointima formation

Eight‐week‐old male C57BL/6 mice were used in this study. Wire‐injured procedure was performed to the right carotid artery as previously described.^18^ In brief, after putting the mouse under anaesthesia, we proceeded to isolate the right internal common carotid artery near the carotid bifurcation, where the right common carotid artery was then denuded and dilated by using a straight guide wire (0.25 mm in diameter). The guide wire was inserted approximately 15 mm from the internal carotid artery, nearing the bifurcation site, then proceeded with five rotational passes through a transverse arteriotomy of the internal carotid artery. Upon leaving the wire inside for 3 min, it was carefully withdrawn and the blood flow was subsequently restored. All post‐procedure recoveries were successful, and no symptoms of stroke were observed. Injured carotid artery segments were harvested for further analysis at designated time points.

Evans blue staining was used to evaluate the degrees of re‐endothelialization. On Day 0, Day 3, Day 7 and Day 14, the mice were sacrificed and perfused with PBS followed by 4% PFA, then injected with Evans blue dye (2%). The right common carotid artery of the mice was harvested and washed with PBS. The surface of non‐endothelialization was marked with blue staining, whereas the area of endothelialization was marked with white. The rate of re‐endothelialization was calculated by dividing the area marked with white to the total area.

### 2.3 Immunofluorescence

Paraffin sections were deparaffinized with xylene and then rehydrated with ethanol. After that, sections were incubated in antigen retrieval buffer at 95°C for 20 min for antigen retrieval, followed by incubation with blocking solution (5% goat serum in PBS) at room temperature for 30 min. Thereafter, the sections were incubated with primary antibodies against CD31 (Mouse IgG, ab9498, 1:200) and PCNA (Rabbit IgG, ab92552, 1:200) at 4°C overnight. The sections were then incubated with goat anti‐Rabbit IgG (H+L) (Alexa Fluor Plus 488, ThermoFisher, A32731, 1:500) and goat anti‐Mouse IgG (H+L) (Alexa Fluor Plus 647, ThermoFisher, A32728, 1:500) at 37°C for 60 min, followed by an incubation with DAPI (4’,6‐diamidino‐2‐phenylindole) for 10 min at room temperature. After mounting, the sections were examined by using fluorescence microscope where images were captured and then processed by Photoshop software (Adobe).

### 2.4 RT‐qPCR

Harvested cells were lysed with TRIzol reagent (Invitrogen), and total RNA was extracted as instructed by standard RNA extraction protocol. Complementary DNA (cDNA) was synthesized from 1 μg of total RNA and random hexamers included in cDNA Synthesis kit (Takara, Japan). Forward and reverse primers were synthesized by Tsingke, China. One microliter of diluted cDNA was used per 10 μl reaction using the TB Green^®^ Fast qPCR Mix (Takara, Japan). Real‐time PCR was performed on a real‐time PCR system (Bio Rad, USA). All reactions were performed in triplicate. Details of the primer sequences are presented in Table 1.

**TABLE 1:** Primer sequence

Gene	Forward primers (5′–3′)	Reverse primers (5′–3′)
Actin	GTGACGTTGACATCCGTAAAGA	GCCGGACTCATCGTACTCC
IMP1	GGCCATCGAAACTTTCTCGG	GCACTTCCCATCGGAGCTG
IMP2	GACTACCCCGACCAGAACTG	GAGGCGGGATGTTCCGAATC
IMP3	TCGAGGCGCTTTCAGGTAAAA	CTCTGCCGTTTAGGGACCG

### 2.5 Western blot

Proteins were extracted using a radioimmunoprecipitation assay (RIPA) buffer (50 mM Tris‐HCL, 150 mM NaCl, 0.1% SDS, 0.25% sodium deoxycholate, 1% NP‐40, 1× protease inhibitor cocktail (sigma), 1× Phosphatase inhibitor (sigma), pH 7.4), and the total protein concentration was determined by using bicinchoninic acid assay (BCA) protein assay kit (Thermo Fisher Scientific). The cell lysate was separated by using sodium dodecyl sulfate (SDS) polyacrylamide gels, transferred to poly (vinylidene fluoride) (PVDF) membrane filter papers and subsequently used for immunoblotting. The washing buffer solution is comprised of 0.1% Tween 20 in Tris‐buffered saline (20 mM Tris and 150 mM NaCl; pH 7.4), and the blocking buffer was made with 5% skim milk (sigma) in washing buffer. A selection of protein markers were examined, including:β‐Actin (CST, #3700, 1:1000), VEGF(CST, #2463, 1:1000), Akt (CST, #4691, 1:1000), p‐Akt(CST, #4060, 1:2000), PI3K(CST, #4257, 1:1000) and p‐PI3K(CST, #17366,1:1000).

### 2.6 Cell culture, transfection and chemical treatments

Human umbilical vein endothelial cells (HUVECs) were purchased from ScienceCell Research Laboratories (USA) and cultured according to the standard guideline. HUVECs were cultured in endothelial cell medium (1001, ScienceCell Research Laboratories) supplemented with 5% foetal bovine serum, endothelial cell growth supplement and antibiotic solution.

Human embryonic kidney 293T cells were purchased from ATCC (Manassas, VA, USA) and maintained in Dulbecco's modified Eagle's medium (DMEM) supplemented with 10% foetal bovine serum (Gibco, North America), 100 U/ml penicillin and 20 U/ml streptomycin. Cells were incubated at 37°C in a humidified chamber containing 5% CO_2_.

The adenovirus‐encoding IMP3 (ad‐IMP3) was constructed and amplified by Boi‐Link (Shanghai, China). An adenovirus‐encoding red fluorescence protein (ad‐RFP) was used as a negative control. HUVECs were transfected with ad‐IMP3 or ad‐RFP (100 pfu number/cell) for 48 h for the following experiments.

Si‐IMP3 was purchased from GenePharma Company (Shanghai, China); a non‐targeting siRNA (GenePharma, Shanghai, China) was used as a negative control. HUVECs were transfected using Lipofectamine 3000 reagent (Invitrogen, USA) according to the manufacturer's instructions.

Axitinib was resuspended in DMSO and HUVECs treated with 0.5 μg/ml Axitinib.^19^

### 2.7 CCK8 and EDU assay

CCK8 assay was performed using CCK‐8 kit (Dojindo, Japan) according to the manual. In brief, differentially treated cells (1 × 10^3^ cell/well) were seeded in 96‐well plates 24 h before the assay. Next, 10 μl CCK8 solution and serum‐free medium (1:10) mixture were added to each well. Cells were then incubated at 37°C for 1–2 h (the end‐point of incubation was determined visually by colour change), and the absorbance at 450 nm was measured by a microplate reader (SpectraMax i3x, Molecular Devices, USA).

Cell proliferation quantification was performed using Cell‐Light EdU DNA Cell Proliferation Kit (Ribobio, China) according to the manufacturer's instructions. In brief, cells were incubated in the dark with 50ul EdU reagents for 2 h, then fixed by 4% paraformaldehyde and permeabilized by 0.5% Triton X‐100. Thereafter, cells were stained by Apollo Dye Solution, and the nucleic acid was stained by Hoechst‐33342. The images were produced by Leica DM2000 Fluorescence Microscope (Leica, Germany) under green fluorescence channel.

### 2.8 TUNEL

TUNEL assay was performed with In Situ Cell Death Detection Kit (Roche) according to the manufacturer's instructions. In brief, cells were washed with PBS, then fixed by 4% PFA, and permeabilized by freshly prepared 0.1% Triton X‐100 in 0.1% sodium citrate. Thereafter, cells were incubated with TUNEL reaction mixture for 60 min at 37°C in a humidified chamber while kept in the dark. After another PBS wash, the nucleic acid was stained by DAPI. The images were produced by Leica DM2000 Fluorescence Microscope (Leica, Germany).

### 2.9 Migration and wound healing

Tanswell^®^ and wound‐healing assay were carried out to evaluate the migration capacity of different treated HUVECs.

Tanswell^®^ chamber (Costar, Boston, MA, USA) was used to carry out cell migration assay. The upper chamber was seeded with HUVECs under different treatments and maintained in serum‐free medium, while the bottom chamber was added with a medium containing 10% FBS. After incubating for 16 h, migrated cells were fixed with 4% PFA and stained with 0.5% crystal violet (Sigma‐Aldrich), followed by visualization and counting under light microscope, three randomly selected field‐of‐view were taken for each sample.

For wound‐healing assay, cells were seeded onto 96‐well plates and allowed to grow into monolayers; the monolayer surface was then scratched using a 200 μl tip when the culture reached approximately 90% confluency. After three PBS washes, cells were incubated for 16 h in culture medium supplemented with 1% FBS. Wound width was visualized and measured under a light microscope.

### 2.10 Assessment of the Stability of VEGF mRNA

HUVECs were infected with ad‐IMP3 or ad‐RFP as described before, then treated with 10 μg/ml alpha‐amanitin (RNA‐polymerase II inhibitor). Total RNA was harvested at 0, 2, 4 and 6 h after alpha‐amanitin treatment. The expression level of VEGF mRNA was evaluated by real‐time RT‐PCR.

### 2.11 Dual luciferase reporter assay

VEGF 5’‐UTR, CDS sequence and 3’‐UTR were amplified and inserted into a pGL4 vector (Promega, USA), and named pGL4‐5’‐UTR, pGL4‐CDS and pGL4‐3’‐UTR, respectively. 293T cells were seeded onto 24‐well plates 24 h before the experiment. Empty vector, pGL4‐3’‐UTR, pGL4‐CDS or pGL4‐5’‐UTR together with AAV2‐IMP3 or AAV2‐Control and Renilla luciferase plasmid were co‐transfected into 293T cells by Lipofectamine 3000 reagent (Invitrogen, USA). Forty‐eight hours post‐transfection, the cells were harvested and lysed. Firefly luciferase activities were detected with a Dual‐Luciferase Reporter Assay System E1910 (Promega, USA) and normalized to control Renilla luciferase levels.

### 2.12 RNA Immunoprecipitation

RNA immunoprecipitation (RIP) was performed with EZ‐Magna RIP RNA‐Binding Protein Immunoprecipitation Kit (Millipore, USA) according to the manufacturer's instructions. In brief, HUVECs were harvested and resuspended in RIP lysis buffer; the cell lysates were incubated overnight with IP buffer containing magnetic beads conjugated with either rabbit anti‐IMP3 antibody (Abcam), normal mouse IgG (Merck Millipore) or Anti‐SNRNP70 antibody (Merck Millipore). Next, the magnetic beads were incubated with proteinase K after washing six times with RIP Wash Buffer. Total RNA was subsequently isolated from the extracts using the TRIzol (Invitrogen, USA).

### 2.13 Statistical analysis

Each dataset was expressed as mean ± SD, which is calculated from data of at least three biological replicates experiments. A two‐sided Student's *t*‐test was used to analyse the differences between two groups. *p* < 0.05 indicates statistical significance. All statistical analysis were performed using SPSS 18.0 and GraphPad Prism 6.

## 3 RESULTS

### 3.1 IMP3 expression is up‐regulated in endothelial cells within injured vascular endothelium

**FIGURE 1:**
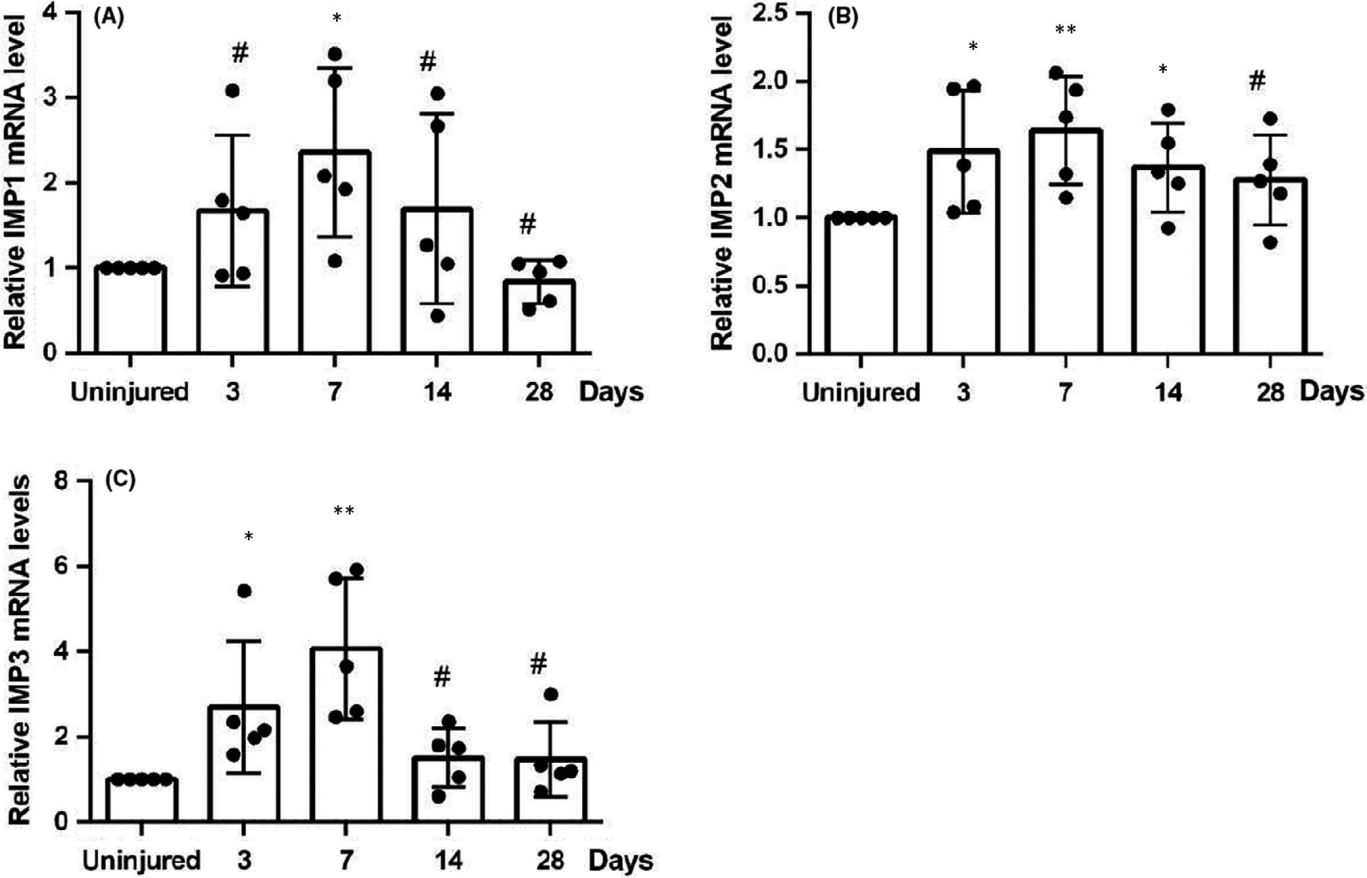
IMPs expression are up‐regulated within lesion after vascular injury. Eight‐week‐old wildtype C57/BL6 mice were divided into two groups—control group that received sham surgery and experiment group that received right carotid artery wire injury surgery; mice were then sacrificed for at destinated time point after receiving sham/wire injury surgery, followed by total RNA extraction from the isolated ECs then qRT‐PCR analyses. Changes in IMP1 (A), IMP2 (B) and IMP3 (C) RNA level at Day 3, 7, 14 and 28 post injury. *n* = 5 per group, the data are presented as mean ± SD, ^#^ *p* > 0.05, *p < 0.05 and **p < 0.01

To determine changes in the mRNA expression levels of IMPs and their potential involvements in endothelial recovery in response to injuries, we introduced arterial injuries by performing wire injury surgery to the right carotid artery of wildtype C57/BL6 mouse. Carotid arteries of C57/BL6 mice were harvested at 3, 7, 14 or 28 days post‐surgery. Harvested arteries were digested and dissociated into single cells, followed by magnetic‐activated cell sorting. Endothelial cells were isolated by using anti‐CD31 conjugated magnetic beads. RT‐qPCR analysis showed the mRNA levels of IMP1 (Figure 1A), IMP2 (Figure 1B) and IMP3 (Figure 1C) at different harvesting time‐ points. Interestingly, we found that IMP1, IMP2 and IMP3 were all up‐regulated at varying degrees in endothelial cells from injured arteries, which was a direct contrast to the uninjured group. Surprisingly, IMP3 was the most up‐regulated out of other IMPs. Upon a closer inspection, the expression level of IMP3 increased from Day 3, peaked on Day 7, and then began to decline. Thus, we speculated that this dynamic expression of IMP3 could imply its potential regulatory role during the process of endothelial recovery.

### 3.2 IMP3 accelerates the progress of re‐endothelialization after arterial injury

**FIGURE 2:**
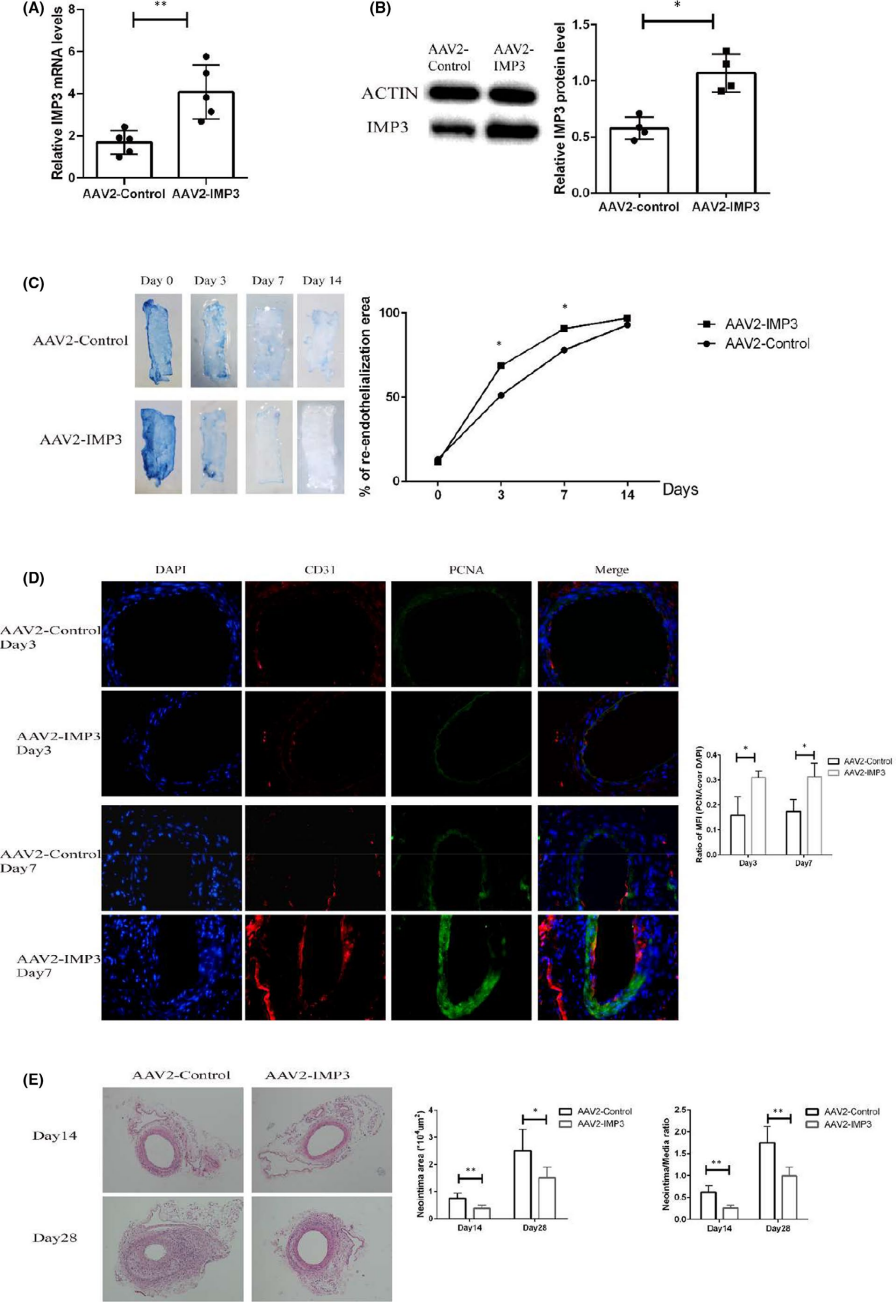
IMP3 accelerates the progress of re‐endothelialization after arterial injury. Mice were sacrificed for the carotid aortas three days after AAV2‐IMP3 or AAV2‐Control injection; both total RNA and total proteins were harvested from the isolated ECs. (A) Change in IMP3 RNA level, *n* = 5 per group. (B) Changes in protein expression, the relative expression of IMP3 was quantified as fold change shown in grey. *n* = 4 per group. Mice were administered with AAV2‐IMP3 or AAV2‐Control injection seven days before carotid artery injury surgery. Mice were sacrificed for the injured carotid arteries at designated time points. Evans staining (C) was performed to estimate the degree of re‐endothelialization on Day 0, Day 4 and Day 7; the injured area was stained by Evans blue. The rate of re‐endothelialization was calculated by dividing the area marked with white to the total area. *n* = 3 per group. (D) Immunofluorescence staining of CD31 and PCNA in mice carotid arteries: CD31 was stained in red whereas PCNA was stained in green, nuclei were stained in blue, relative mean fluorescence intensity (MFI) of PCNA over DAPI signal were statistically analysed, *n* = 3 per group. (E) HE staining was used to estimate the degree of neointimal hyperplasia on Day 14 and Day 28, the neointima area and neointima‐to‐media area ratios were statistically analysed. *n* = 5 per group. The data are presented as mean ± SD. *p < 0.05 and **p < 0.01

The up‐regulation of IMP3 in endothelium after carotid artery injury suggested a possible contributing role of IMP3 during the process of re‐endothelialization after arterial injury. To elaborate, we overexpressed IMP3 in Wild type C57BL/6 by AAV2‐IMP3 injection seven days before the surgery, and AAV2‐Control injection was used as a negative control. Some of the mice were sacrificed for their carotid aortas three days after AAV2‐IMP3 or AAV2‐Control injection, then total RNA and protein were harvested from the isolated ECs. Results from RT‐qPCR and Western blot analysis confirmed that the expression of IMP3 was up‐regulated after three days of AAV2‐IMP3 injection (Figure 2A,B). We also thoroughly examined other organs such as heart, liver and kidney, and found no substantial side effects (Figure S1). Mice were sacrificed for carotid arteries collection at 3, 7, 14 or 28 days after the surgery. The extent of re‐endothelialization were quantified by Evans blue staining of the injury site. (Figure 2C). Carotid artery endothelium of AAV2‐IMP3 injection mice and AAV2‐Control injection mice was both severely denuded immediately after surgery. However, we found that injured endothelium from AAV2‐IMP3 injection mice demonstrated a more robust recovery when compared with AAV2‐Control injection mice. Immunofluorescence also demonstrated that the lesions of IMP3 overexpressing mice have larger numbers of CD31‐positive cells on Day 3 and Day 7 (Figure 2D). In addition, CD31‐positive endothelial cells expressed higher level of PCNA (Figure 2D), which could indicate that a high level of IMP3 expression can promote EC proliferation. Subsequent neointimal hyperplasia was evaluated at 14, 28 days after the surgery (Figure 2E), to which our results showed a significant decline in the injured arteries of IMP3 overexpressed mice.

Collectively, these results demonstrated that overexpression of IMP3 could accelerate the process of re‐endothelialization, whilst attenuating the progression of neointimal hyperplasia.

### 3.3 IMP3 enhances endothelial cell proliferation but represses apoptosis

**FIGURE 3:**
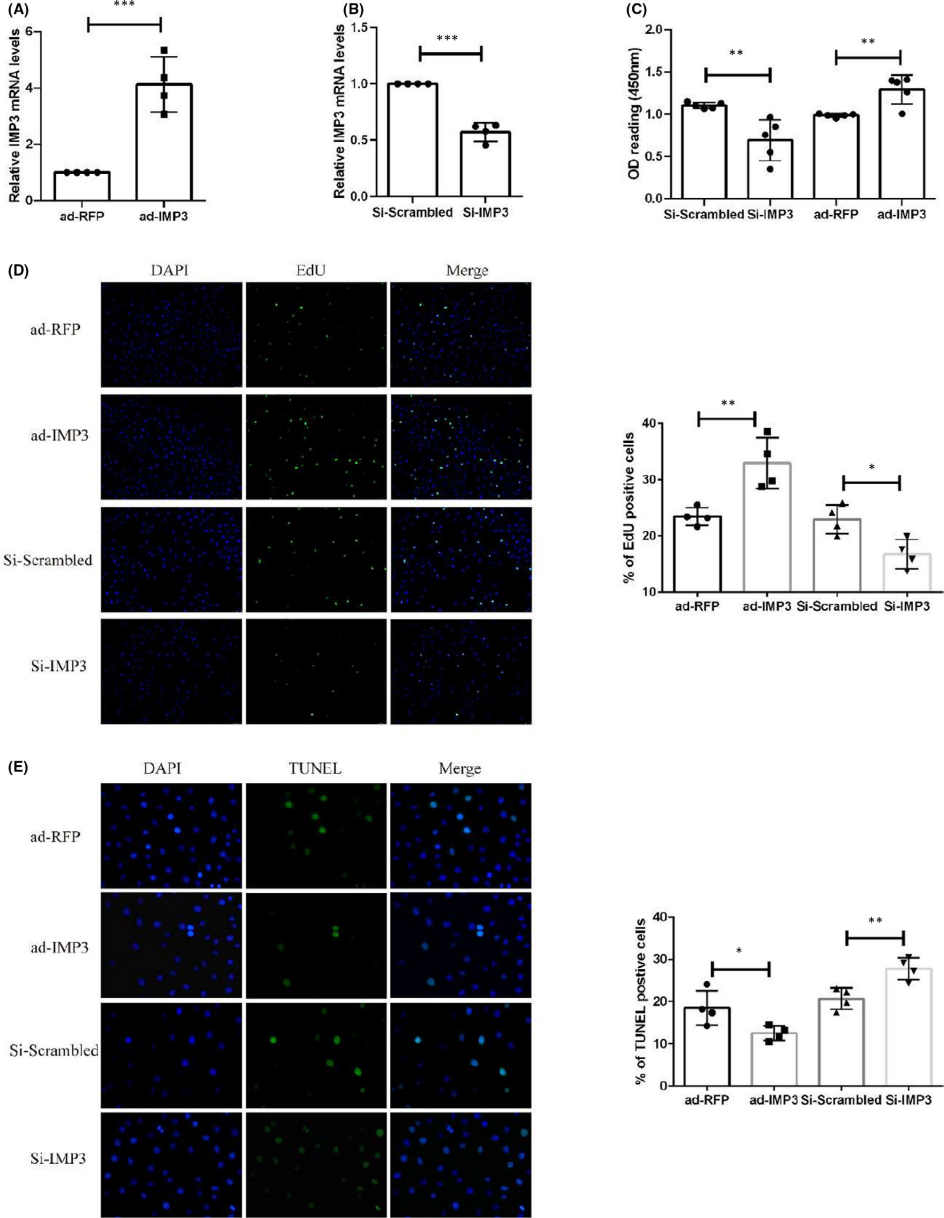
IMP3 enhances endothelial cell proliferation while represses apoptosis. IMP3 RNA levels were measured by qRT‐PCR analysis to validate transfection efficiency of ad‐IMP3 (A) and Si‐IMP3 (B), *n* = 4 per group. HUVECs were subjected to ad‐IMP3, ad‐RFP, Si‐IMP3 or Si‐Vehicle treatment, and followed by different analysis. (C) CCK‐8 assay was used to access cell viability, *n* = 5 per group. (D) EDU assay was performed to access cell proliferation, *n* = 4 per group. (E)TUNEL assay was used for apoptosis, *n* = 4 per group. Experiments were repeated and the data are presented as mean ± SD. *p < 0.05, **p < 0.01 and ***p < 0.001

Amongst the numerous functions of vascular endothelial cells, endothelial proliferation and migration are pivotal in re‐endothelializing injured arterial endothelium. Thus, in order to determine the role of IMP3 expression in endothelial cell proliferation and migration, we sought to modulate IMP3 expression by ad‐IMP3 transfection. After several trials, we established appropriate induction time and concentration of ad‐IMP3 to maximize transfection efficiency, which ensured a robust up‐regulation of IMP3 expression (Figure 3A). Si‐IMP3 was also employed for the purpose of downregulating IMP3 expression in contrast to Si‐Scrambled (Figure 3B). We performed CCK8 and EdU assays to assess the rate of endothelial cell proliferation under IMP3 overexpression or knock‐down. As shown by the results of CCK8 assays, IMP3 overexpression significantly promoted the viability of endothelial cell in culture, whilst IMP3 knock‐down decreased endothelial cell viability (Figure 3C). EdU staining data showed markedly increased red fluorescence ratio in cells overexpressing IMP3, indicating higher proliferative capacity (Figure 3D), whereas the knock‐down effect by Si‐IMP3 elicited an opposite effect (Figure 3C,D). Moreover, TUNEL assays revealed a negative correlation between increased IMP3 expression and endothelial cell apoptosis (Figure 3E).

Our results demonstrated that IMP3 enhances endothelial cell proliferation while repressing apoptosis in vitro.

### 3.4 IMP3 promotes endothelial cell migration

**FIGURE 4:**
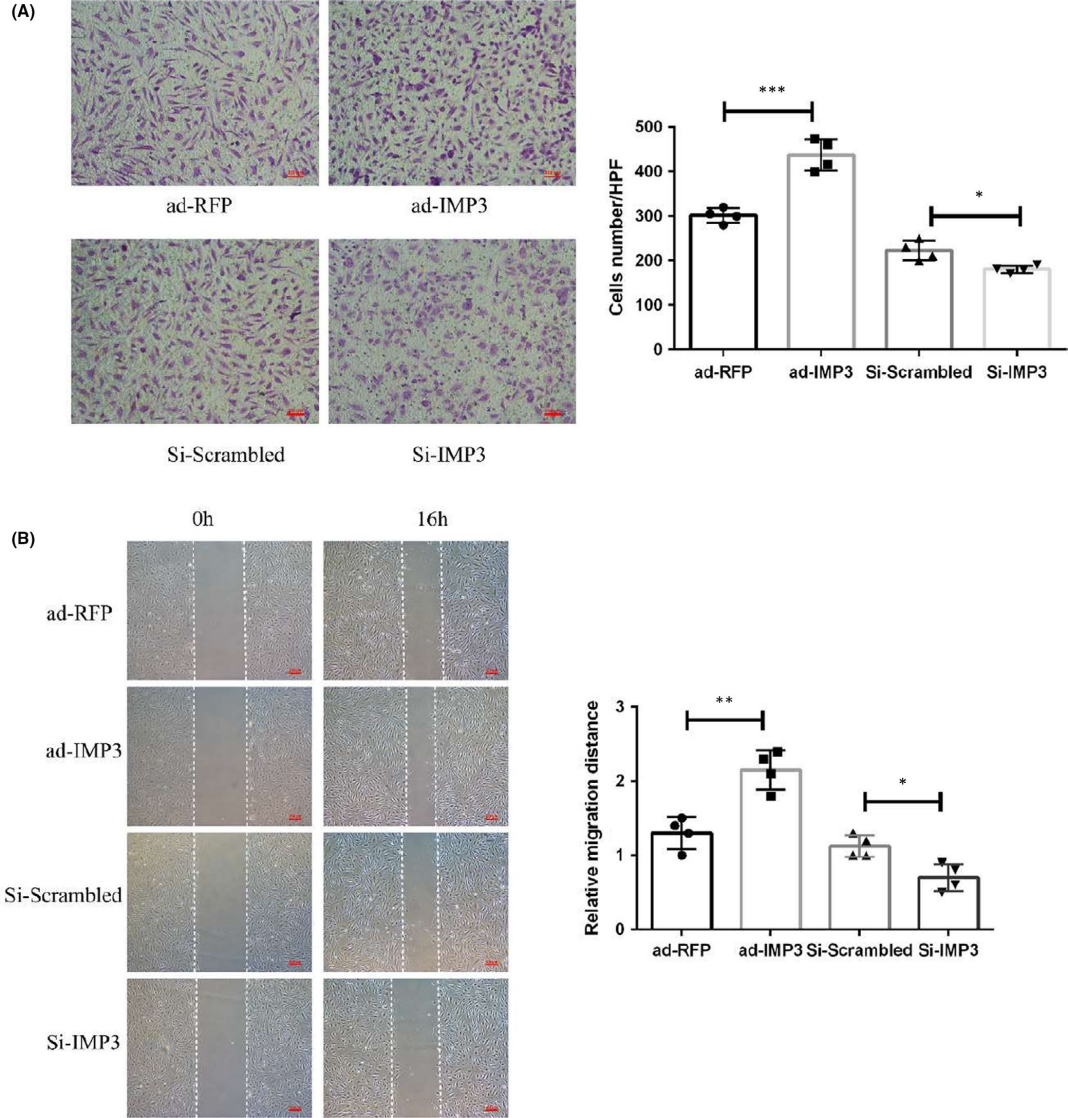
IMP3 promotes endothelial cell migration. HUVECs were subjected to ad‐IMP3, ad‐RFP, Si‐IMP3 or Si‐Vehicle treatment, and followed by different analysis. (A) Representative images of transwell assay at 16 h after overexpression or knock‐down of IMP3. Migrated cells were counted and calculated from the average cell number of three randomly chosen fields. (B) Wound‐healing assays were employed for accessing cell migration ability. Cell migration was observed at 16h post‐wounding (dotted line indicates wound edge). The mean distance migrated by the HUVECs was quantified. Experiments were repeated four times and the data are presented as mean ± SD. (*n* = 4). *p < 0.05 and **p < 0.01

Sequentially, cell migration assays were performed by Transwell and scratch wound assays. Both assays later revealed that overexpression of IMP3 could significantly promote endothelial cell migration (Figure 4A,B). In contrast, knock‐down of IMP3 considerably impaired the migratory capacity of endothelial cell (Figure 4A,B).

### 3.5 IMP3 regulates endothelial cell functions through activation of VEGF/PI3K/Akt signalling pathway

**FIGURE 5:**
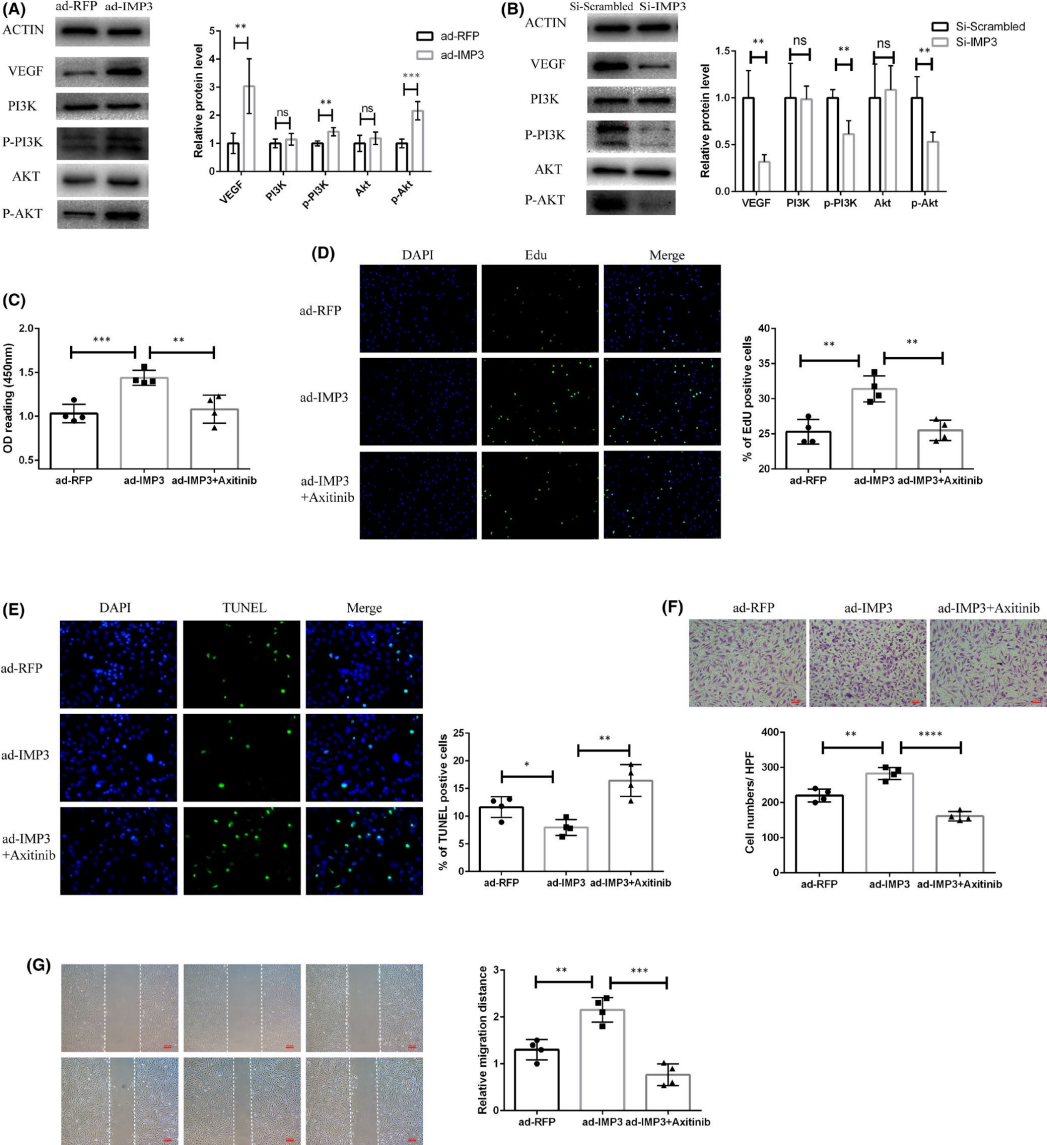
IMP3 regulates endothelial cell functions through activation of VEGF/PI3K/Akt signalling pathway. (A) After transfection of ad‐IMP3 or ad‐RFP, protein level of VEGF, PI3K, PPI3K and Akt in transfected HUVECs were measured by Western blot. ACTIN was selected as a protein control; the relative expression of proteins were quantified as fold change shown in grey (*n* = 3). (B) After HUVECs were transfected by Si‐Vehicle or Si‐IMP3, the protein expression level of VEGF, PI3K, PPI3K and Akt were measured by western blot. ACTIN was used as a protein control; the relative expression of protein was quantified as fold change shown in grey. HUVECs were subjected to ad‐IMP3, ad‐RFP or ad‐IMP3+Axitinib (0.5 μg/ml) treatment, followed by various analysis. (C) CCK‐8 assay was used to measure cell viability. (D) EDU assay was used to measure cell proliferation. (E) TUNEL assay was used to measure apoptosis. (F) Transwell assay and (G) wound‐healing assay were employed for measuring cell migration ability. Experiments were repeated four times and the data are presented as mean ± SD. (*n* = 4). ^#^ *p* > 0.05,*p < 0.05, **p < 0.01 and ***p < 0.001

Previous studies have revealed that activation of the VEGF/PI3K/Akt pathway was crucial for re‐endothelialization and endothelial cell activation,^20^, ^21^ where the activated endothelial cells exhibit enhanced cell survival, proliferation and migration. To elucidate the underlying mechanisms of IMP3, we analysed protein expression level of VEGF, PI3K, p‐PI3K, Akt and p‐Akt by Western blotting. The results showed that overexpression of IMP3 could contribute to an increase in VEGF, p‐PI3K and p‐Akt protein expression (Figure 5A), whilst decreasing IMP3 expression achieved opposite effects (Figure 5B). Previous results have indicated that IMP3 could increase proliferative and migratory capacity of endothelial cells whilst inhibiting apoptosis, whereas these effects were partially revoked in endothelial cells treated with VEGFR‐specific inhibitor Axitinib. CCK8 assays showed that the overall cell viability of HUVECs was partially suppressed by Axitinib (Figure 5C); similarly, after treatments with Axitinib, the percentage of EdU‐positive cells decreased drastically (Figure 5D). Moreover, the results of TUNEL assays revealed that Axitinib could abrogate the anti‐apoptotic effects of IMP3 overexpression, which was observed in ad‐IMP3‐transfected endothelial cells (Figure 5E). Lastly, as shown in both transwell and wound‐healing assays, Axitinib treatment also reversed the enhancements in cell migratory functions induced by overexpressing IMP3 (Figure 5F–G).

Collectively, these results suggested that IMP3 could not promote endothelial cells proliferation, migration or represses apoptosis independently of the VEGF/PI3K/Akt signalling pathway.

### 3.6 IMP3 as RNA‐binding protein increases the stability of VEGF mRNA

**FIGURE 6:**
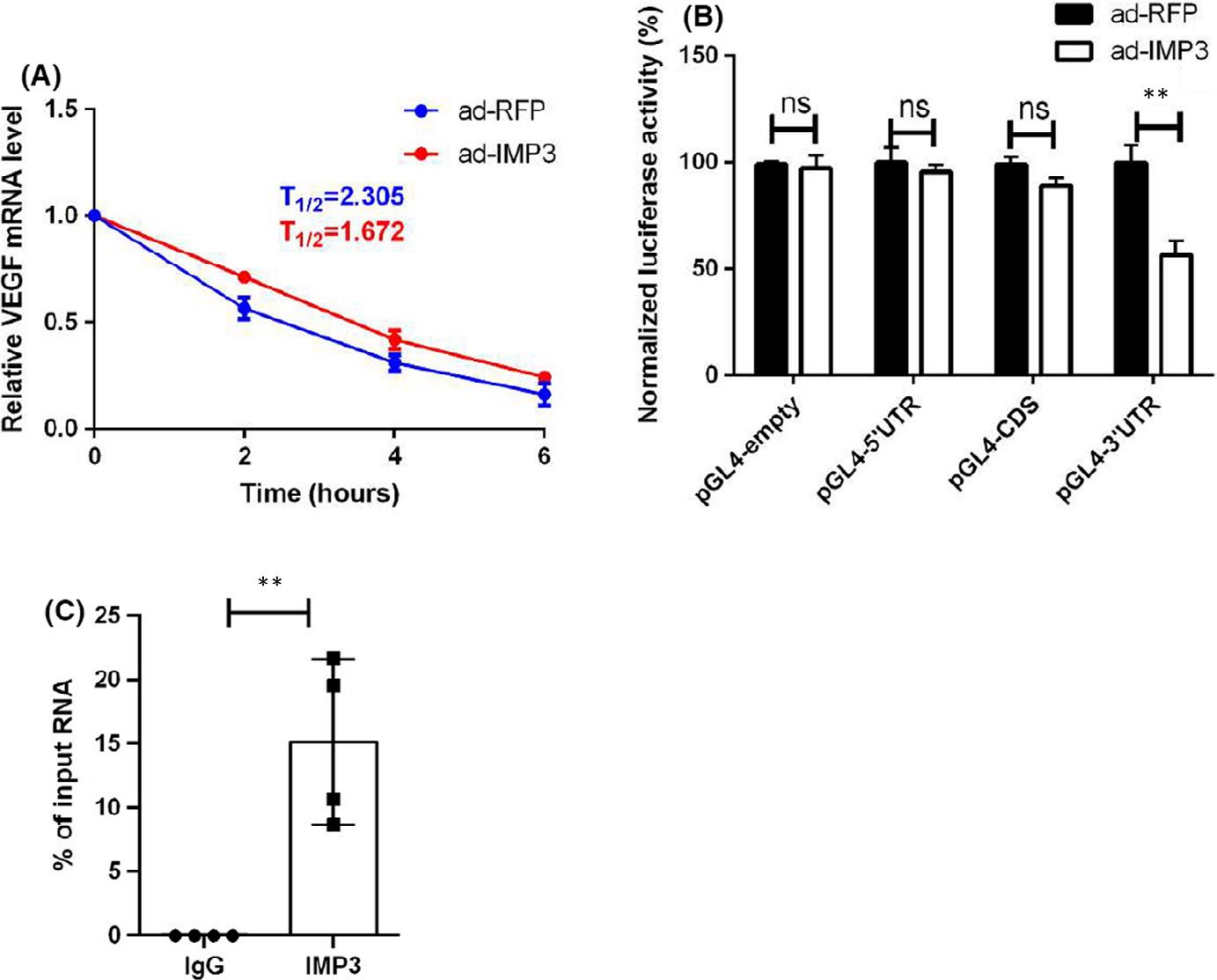
IMP3 as RNA‐binding protein increases stability of VEGF mRNA. (A) HUVECs were subjected to ad‐IMP3 or ad‐RFP treatment prior to alpha‐amanitin treatment; the total RNA was harvested at designated time points. mRNA expressions were analysed by qRT‐PCR, half‐life was calculated accordingly, *n* = 3 per group. (B) Dual‐luciferase reporter assay was performed after co‐transfection with reporter vector and ad‐IMP3 or ad‐RFP in 293T for 48 h, *n* = 3 per group. (C) HUVECs were harvested and anti‐IMP3 antibody was used to extract IMP3 protein and its associated RNA molecules via immunoprecipitation. IgG beads were used as negative control. RIP products were analysed by RT‐qPCR, *n* = 4 per group. Experiments were repeated and the data are presented as mean ± SD. **p < 0.01

IMP3 is a well‐known RNA‐binding protein that participates in post‐transcriptional modification via regulating stability of its target RNAs.^7^, ^22^, ^23^ Naturally, we sought to investigate whether or not IMP3 is capable of regulating VEGF mRNA stability. To elaborate, we induced IMP3 overexpression or knocked down in order to observe changes in VEGF mRNA levels, whilst under alpha‐amanitin treatment (RNA‐polymerase II inhibitor). RT‐qPCR data revealed a strong reinforcement of VEGF mRNA stability by IMP3 (Figure 6A). Previous studies have also reported that the RNA‐recognition site for IMP3 is likely to be located in the coding regions or 3’‐UTRs of target transcripts.^23^, ^24^ To verify that VEGF is a direct target of IMP3, we constructed 5’‐UTR, coding sequence and 3’‐UTR of VEGF mRNA (pGL4‐5’‐UTR, pGL4‐CDS and pGL4‐3’‐UTR) and empty (pGL4‐empty) luciferase reporter vector. Luciferase assay showed that IMP3 significantly promoted luciferase activity of pGL6‐3’‐UTR and exclusively so used (Figure 6B). Finally, to further determine whether or not a direct interaction between IMP3 and VEGF mRNA was present, RNA immunoprecipitation was performed in endothelial cell with IMP3 antibody or IgG control. RIP results revealed that VEGF mRNA was preferentially enriched in immunoprecipitation against IMP3 antibody compared with IgG control (Figure 6C).

## 4 DISCUSSION

The present study identified a novel functional role of IMP3 in endothelial cell biology. Our current research demonstrated that RNA‐binding protein IMP3 could be critically involved in re‐endothelialization and prevention of neointimal lesion formation after arterial injury, where IMP3 exerts its regulatory functions via targeting the VEGF/PI3K/Akt signalling pathway.

Coronary angioplasty and stents implantation are universally accepted in the clinics and often considered the most effective treatments for patients who suffer from obstructive coronary artery disease. However, restenosis often occurs as a result of angioplasty or stent implantation, and it has been considered a pressing challenge in coronary interventional treatment.^25^ A retrospective study conducted by Moussa report that approximately 1 in 10 interventions in the United States are for in‐stent restenosis.^26^ Although many clinical trials have reported that the use of next‐generation DES reduced in‐stent restenosis at an early stage,^1^, ^27^ the overall landscape of preventative strategies for ISR has remained relatively unchanged over the years.^26^

IMP3, also known as insulin‐like growth factor 2 (IGF2) mRNA‐binding protein 3 (IGF2BP3), is a member of the insulin‐like growth factor 2 mRNA‐binding proteins family.^28^, ^29^, ^30^ IMPs play its role in post‐translational RNA processing, such as transcript localization, translation and stabilization^31^, ^32^, ^33^; additionally, IMPs family were recently identified as a family of m6A readers.^8^, ^34^ However, previous research mainly focussed on the biological functions of IMPs in tumours and embryogenesis. For example, IMP3 participates in RNA trafficking and stabilization which promotes cell proliferation, migration in tumour progression and metastasis.^35^, ^36^ The proliferation and migration capacity of endothelial cells is critical in vascular re‐endothelialization process and plays an important role in in‐stent restenosis.^37^, ^38^, ^39^ However, the role of IMPs in endothelium and re‐endothelialization has not yet been clarified. Therefore, we surgically introduced wire injuries to the right carotid artery of wildtype C57/BL6 mice, which in turn, triggered endothelial recovery as a response to vascular injuries; we then screened the expressions of three members of IMPs family (IMP1, IMP2 and IMP3). Interestingly, we found that IMPs expressions were all elevated during specific stages of endothelial recovery, especially IMP3 being the highest of them all. More specifically, the expression level of IMP3 started to increase from Day 3, reaching the highest level on Day 7, followed by a plateau and then declined to the baseline. This shifting pattern of IMP3 expression after introduction of carotid artery injuries was consistent with the progress of re‐endothelialization,^40^ indicating that IMP3 may be involved in the re‐endothelialization process after carotid artery injury.

However, IMP3 is reported to predominantly express in tumour tissues and in foetal tissues.^41^, ^42^ Numerous studies have identified that IMP3 tends to promote tumour cell proliferation, migration and most often associates with poor prognosis.^30^, ^43^ There were a number of articles reported that IMP3 is expressed in many developing human tissues such as the epithelium, placenta and muscles, but not expressed in normal adult tissues.^44^, ^45^ Nevertheless, a study conducted by Bell et al., which analysed the expression of IGF2BPs in various adult mouse tissues by semi‐quantitative RT‐PCR, has found that the expression of IGF2BP3 was largely age‐independent, although modest expressions were observed in the lung, spleen, kidney and gut of 16‐week‐old male mice.^7^ In addition, a study for acute liver failure has reported that in healthy mouse liver, only a small population of cells expressing IMP3 were identified by immunohistochemistry. However, hepatocyte‐like IMP3(+) cells emerged by 24 h after partial hepatectomy; the number of these cells peaked at 48 h after partial hepatectomy and then steadily declined to near basal levels by 96 h after partial hepatectomy.^46^ Due to this feature of IMP3, we focussed on the effect of IMP3 on endothelial cell function and explored its role in re‐endothelialization post injury. According to our available results, endothelial cells express IMP3 at a relatively low level in the uninjured condition; upon receiving wire‐injured surgery, expression level of IMP3 in endothelial cells followed a trend of early elevation and late decrease. This is highly consistent with the pathological changes of the disease.^37^, ^47^ During the early stage of injury, in response to the loss of large number of endothelial cells, the remaining endothelial cells begin to enter a state of proliferation, by up‐regulating the expression of IMP3. Such effect reaches its peak at Day 14; when the endothelial repair process has been largely completed, the demand for cell proliferation is also reduced progressively. Therefore, the expression of IMP3 starts to decline. Notably, a recent publication reported that Gene Set Enrichment Analysis (GSEA) using process networks from Metacore Pathway Analysis showed that knock‐down of IMP3 in human endothelial cell lines (HUVECs) resulted in the downregulation of cell proliferation‐associated proteins such as cyclin B, securin, PBK, CDCA1, SPBC5, HEC, CENP‐A and MAD2a,^48^ which also supports our interpretations from another point of view.

In order to confirm the role of IMP3 in modulating re‐endothelialization after carotid artery injury, we employed AAV2‐IMP3 infection to C57/BL6 mice—mice that were used to establish carotid artery injury model. We found that AAV2‐IMP3 injection mice demonstrated a more robust recovery capability and reduced neointimal hyperplasia compared with AAV2‐Control mice. However, we still lacked information regarding the re‐endothelialization process after knock‐down or knockout IMP3 gene in mice. Despite this limitation, the significant recovery events observed in the injured arteries of AAV2‐IMP3 injection mice does imply that overexpression of IMP3 could be exploited and expanded to promote the repair of artery damage. More importantly, AAV2‐IMP3‐treated mice did not show signs of adverse effects to other organs or tissue toxicity. However, we have not yet investigated the effects of different virus doses in relation to the degree of re‐endothelialization. If possible, we will further explore the optimal dose of virus delivery and investigate if the progress of re‐endothelialization could be enhanced with no additional adverse effects to other organs.

Activation of re‐endothelialization after arterial injury is known to attenuate neointima formation, which is favoured as a new therapeutic strategy for improving endothelial recovery after vascular injuries induced by percutaneous coronary interventions (PCI) procedures.^49^, ^50^ Here, our results could mark the discovery of a novel therapeutic approach, whereby the delivery of target‐specific modulators of IMP3 expression, such as AAV2 package, could be used as a protective agent after stent implantation to promote re‐endothelialization and attenuate stent restenosis.

Activation of endothelial migration and proliferation is believed to be related to re‐endothelialization.^51^, ^52^ Therefore, we conducted in vitro experiments to determine endothelial cell‐specific functions of IMP3. Surprisingly, IMP3 enhances endothelial cell proliferation, migration whilst repressing apoptosis, which was consistent with murine phenotypes. Since our murine experiments did not observe any IMP3‐induced intimal thickening, we did not identify any potential effects of IMP3 in smooth muscle cells.

Present studies revealed that the activation of the VEGF/PI3K/Akt pathway was crucial for re‐endothelialization and activation of endothelial cells.^53^, ^54^ We found that overexpression of IMP3 upregulates the protein expression levels of VEGF, p‐PI3K and p‐Akt, while inhibition of IMP3 inhibits them. Furthermore, VEGFR inhibitor Axitinib abolished the endothelial cell activation of IMP3 overexpression. Collectively, these results suggested a possible mechanism of IMP3‐mediated re‐endothelialization, where IMP3 is the upstream signalling mediator of VEGF/PI3K/Akt pathway. Previous studies have reported that the PI3K pathway is activated by IMP3 in glioblastoma^55^ and prostate cancer tissues,^29^ which is consistent with the role of IMP3 we found in endothelial cells. In addition, some studies have also indicated the interaction between IMP3 and VEGF, for example, Yang et demonstrated that IMP3 knock‐down leads to repressed expression and stability of VEGF mRNA via reading m6A modification^48^; on the other hand, a study conducted by Gharib also reported that there was a significant correlation between VEGF and IMP3 mRNA in lung adenocarcinomas.^56^ Furthermore, a study conducted by Kong showed that both the expressions of IMP3 and VEGF in osteosarcoma tissues were higher than that in adjacent tissues.^57^ Therefore, we could speculate that IMP3 activates the PI3K pathway by stabilizing endogenous VEGF mRNA in endothelial cells. So far, the roles of IMP3 as an RNA‐binding protein was mainly studied due to its ability to recognize and stabilize target mRNA, such as insulin‐like growth factor 2 (IGF2)^58^ or a few other well‐known mRNAs (MYC and CD44).^59^, ^60^ Here, we demonstrated a direct interaction between IMP3 and VEGF mRNA. We observed an increased half‐life of VEGF mRNA transcripts and, consequently, elevated luciferase activity in control of IMP3. And VEGF transcripts were precipitated with IMP3 antibody. Though much work has gone into studying the intricate role of IMPs in mRNA stabilization, its precise mechanism of action still remains unclarified and would require further investigations. A recent study has shown that the KH domains of IMPs family can confer sequence specific recognition of RNAs through their variable loop regions.^58^ If possible, our future experiments will focus on characterizing the specific domains of IMP3 protein that play the essential role in interacting with VEGF mRNA.

**FIGURE 7:**
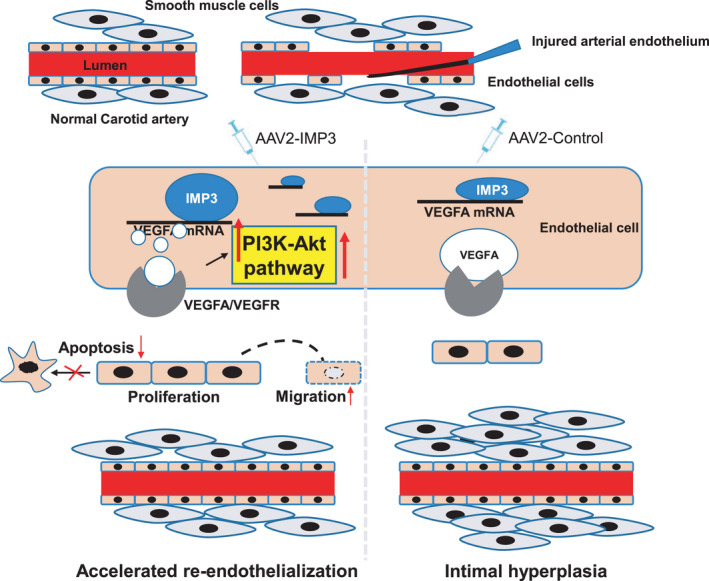
IMP3 regulates re‐endothelialization after vascular injury. The vascular endothelium is denudated after the right carotid artery wire injury surgery. IMP3 could directly bind to VEGF mRNA whilst increasing VEGF mRNA stability, which in turn regulates endothelial cell functions through the activation of VEGF/PI3K/Akt signalling pathway. Overexpression of IMP3 promotes ECs proliferation within the lesion area, enhances ECs migration from adjacent lesion site and inhibits EC apoptosis. Collectively, IMP3 could contribute to accelerated re‐endothelialization as well as reduced intimal hyperplasia

Collectively, we described a novel role of IMP3 as a key player in the process of re‐endothelialization in response to arterial injury. Mechanistically, we demonstrated that overexpression of IMP3 could improve proliferative and migratory capabilities of endothelial cells, and it is responsible for increased re‐endothelialization capacity of endothelial cells after arterial injury. Especially with regard to the absent systemic side effects, the delivery of AAV2‐IMP3 for the regeneration of the endothelium could be a promising therapeutic strategy for improving re‐endothelialization after balloon angioplasty or stent implantation (Figure 7).

## CONFLICT OF INTEREST

The authors confirm that there are no conflicts of interest.

## AUTHOR CONTRIBUTIONS

**Xinmiao Zhou:** Data curation (lead); Formal analysis (lead); Methodology (lead); Validation (lead); Writing – original draft (equal); Writing – review & editing (lead). **Qingqing Ye:** Investigation (equal); Methodology (equal); Writing – review & editing (equal). **Jinlei Zheng:** Project administration (equal). **Lin Kuang:** Methodology (equal); Project administration (equal). **Jianhua Zhu:** Funding acquisition (equal); Resources (equal); Supervision (equal). **Hui Yan:** Conceptualization (equal); Resources (lead); Supervision (equal).

## Supporting information

Figure S1

## Data Availability

Data available on request from the authors.

## References

[1] Stone GW , Ellis SG , Cox DA , et al. A polymer‐based, paclitaxel‐eluting stent in patients with coronary artery disease. N Engl J Med. 2004;350:221‐231. 14724301 10.1056/NEJMoa032441

[2] Piccolo R , Bonaa KH , Efthimiou O , et al. Drug‐eluting or bare‐metal stents for percutaneous coronary intervention: a systematic review and individual patient data meta‐analysis of randomised clinical trials. Lancet. 2019;393:2503‐2510. 31056295 10.1016/S0140-6736(19)30474-X

[3] Stettler C , Wandel S , Allemann S , et al. Outcomes associated with drug‐eluting and bare‐metal stents: a collaborative network meta‐analysis. Lancet. 2007;370:937‐948. 17869634 10.1016/S0140-6736(07)61444-5

[4] Thiel WH , Esposito CL , Dickey DD , et al. Smooth muscle cell‐targeted rna aptamer inhibits neointimal formation. Mol Ther. 2016;24:779‐787. 26732878 10.1038/mt.2015.235 PMC4886937

[5] Curcio A , Torella D , Indolfi C . Mechanisms of smooth muscle cell proliferation and endothelial regeneration after vascular injury and stenting: approach to therapy. Circ J. 2011;75:1287‐1296. 21532177 10.1253/circj.cj-11-0366

[6] Degrauwe N , Suva ML , Janiszewska M , Riggi N , Stamenkovic I . IMPs: an RNA‐binding protein family that provides a link between stem cell maintenance in normal development and cancer. Genes Dev. 2016;30:2459‐2474. 27940961 10.1101/gad.287540.116 PMC5159662

[7] Bell JL , Wachter K , Muhleck B , et al. Insulin‐like growth factor 2 mRNA‐binding proteins (IGF2BPs): post‐transcriptional drivers of cancer progression? Cell Mol Life Sci. 2013;70:2657‐2675. 23069990 10.1007/s00018-012-1186-z PMC3708292

[8] Huang H , Weng H , Sun W , et al. Recognition of RNA N(6)‐methyladenosine by IGF2BP proteins enhances mRNA stability and translation. Nat Cell Biol. 2018;20:285‐295. 29476152 10.1038/s41556-018-0045-z PMC5826585

[9] Dai N , Zhao L , Wrighting D , et al. IGF2BP2/IMP2‐Deficient mice resist obesity through enhanced translation of Ucp1 mRNA and Other mRNAs encoding mitochondrial proteins. Cell Metab. 2015;21:609‐621. 25863250 10.1016/j.cmet.2015.03.006 PMC4663978

[10] Regue L , Minichiello L , Avruch J , Dai N . Liver‐specific deletion of IGF2 mRNA binding protein‐2/IMP2 reduces hepatic fatty acid oxidation and increases hepatic triglyceride accumulation. J Biol Chem. 2019;294:11944‐11951. 31209109 10.1074/jbc.RA119.008778 PMC6682725

[11] Conway A , Van Nostrand E , Pratt G , et al. Enhanced CLIP uncovers IMP protein‐RNA targets in human pluripotent stem cells important for cell adhesion and survival. Cell Rep. 2016;15:666‐679. 27068461 10.1016/j.celrep.2016.03.052 PMC4839292

[12] Hansen TV , Hammer NA , Nielsen J , et al. Dwarfism and impaired gut development in insulin‐like growth factor II mRNA‐binding protein 1‐deficient mice. Mol Cell Biol. 2004;24:4448‐4464. 15121863 10.1128/MCB.24.10.4448-4464.2004 PMC400488

[13] Nishino J , Kim S , Zhu Y , Zhu H , Morrison SJ . A network of heterochronic genes including Imp1 regulates temporal changes in stem cell properties. eLife. 2013;2:e00924. 24192035 10.7554/eLife.00924 PMC3817382

[14] Muller S , Bley N , Glass M , et al. IGF2BP1 enhances an aggressive tumor cell phenotype by impairing miRNA‐directed downregulation of oncogenic factors. Nucleic Acids Res. 2018;46:6285‐6303. 29660014 10.1093/nar/gky229 PMC6158595

[15] Meng L , Lin H , Zhang J , et al. Doxorubicin induces cardiomyocyte pyroptosis via the TINCR‐mediated posttranscriptional stabilization of NLR family pyrin domain containing 3. J Mol Cell Cardiol. 2019;136:15‐26. 31445005 10.1016/j.yjmcc.2019.08.009

[16] Wang Z , Cui M , Shah AM , et al. Mechanistic basis of neonatal heart regeneration revealed by transcriptome and histone modification profiling. Proc Natl Acad Sci USA. 2019;116:18455‐18465. 31451669 10.1073/pnas.1905824116 PMC6744882

[17] Nguyen MA , Karunakaran D , Geoffrion M , et al. Extracellular vesicles secreted by atherogenic macrophages transfer microRNA to inhibit cell migration. Arterioscler Thromb Vasc Biol. 2018;38:49‐63. 28882869 10.1161/ATVBAHA.117.309795 PMC5884694

[18] Xiao Q , Zhang F , Grassia G , et al. Matrix metalloproteinase‐8 promotes vascular smooth muscle cell proliferation and neointima formation. Arterioscler Thromb Vasc Biol. 2014;34:90‐98. 24158518 10.1161/ATVBAHA.113.301418

[19] Siedlecki J , Wertheimer C , Wolf A , et al. Combined VEGF and PDGF inhibition for neovascular AMD: anti‐angiogenic properties of axitinib on human endothelial cells and pericytes in vitro. Graefe's Arch Clin Exp Ophthalmol. 2017;255(5):963‐972. 28161830 10.1007/s00417-017-3595-z

[20] Wang H , Yin Y , Li W , et al. Over‐expression of PDGFR‐beta promotes PDGF‐induced proliferation, migration, and angiogenesis of EPCs through PI3K/Akt signaling pathway. PLoS One. 2012;7:e30503. 22355314 10.1371/journal.pone.0030503 PMC3280261

[21] Ackah E , Yu J , Zoellner S , et al. Akt1/protein kinase Balpha is critical for ischemic and VEGF‐mediated angiogenesis. J Clin Invest. 2005;115:2119‐2127. 16075056 10.1172/JCI24726 PMC1180542

[22] Schmiedel D , Tai J , Yamin R , Berhani O , Bauman Y , Mandelboim O . The RNA binding protein IMP3 facilitates tumor immune escape by downregulating the stress‐induced ligands ULPB2 and MICB. eLife. 2016;5:e13426. 26982091 10.7554/eLife.13426 PMC4805531

[23] Palanichamy JK , Tran TM , Howard JM , et al. RNA‐binding protein IGF2BP3 targeting of oncogenic transcripts promotes hematopoietic progenitor proliferation. J Clin Invest. 2016;126:1495‐1511. 26974154 10.1172/JCI80046 PMC4811152

[24] Schneider T , Hung L‐H , Aziz M , et al. Combinatorial recognition of clustered RNA elements by the multidomain RNA‐binding protein IMP3. Nat Commun. 2019;10:2266. 31118463 10.1038/s41467-019-09769-8 PMC6531468

[25] Huynh T , Perron S , O'Loughlin J , et al. Comparison of primary percutaneous coronary intervention and fibrinolytic therapy in ST‐segment‐elevation myocardial infarction: bayesian hierarchical meta‐analyses of randomized controlled trials and observational studies. Circulation. 2009;119:3101‐3109. 19506117 10.1161/CIRCULATIONAHA.108.793745

[26] Moussa ID , Mohananey D , Saucedo J , et al. Trends and outcomes of restenosis after coronary stent implantation in the United States. J Am Coll Cardiol. 2020;76:1521‐1531. 32972528 10.1016/j.jacc.2020.08.002

[27] Windecker S , Serruys PW , Wandel S , et al. Biolimus‐eluting stent with biodegradable polymer versus sirolimus‐eluting stent with durable polymer for coronary revascularisation (LEADERS): a randomised non‐inferiority trial. Lancet. 2008;372:1163‐1173. 18765162 10.1016/S0140-6736(08)61244-1

[28] Okabayshi M , Kataoka TR , Oji M , et al. IGF2BP3 (IMP3) expression in angiosarcoma, epithelioid hemangioendothelioma, and benign vascular lesions. Diagn Pathol. 2020;15:26. 32293476 10.1186/s13000-020-00951-x PMC7087384

[29] Zhang X , Wang D , Liu B , et al. IMP3 accelerates the progression of prostate cancer through inhibiting PTEN expression in a SMURF1‐dependent way. J Exp Clin Cancer Res. 2020;39:190. 32938489 10.1186/s13046-020-01657-0 PMC7493339

[30] Tschirdewahn S , Panic A , Püllen L , et al. Circulating and tissue IMP3 levels are correlated with poor survival in renal cell carcinoma. Int J Cancer. 2019;145:531‐539. 30650187 10.1002/ijc.32124

[31] Zhang Y , Zhao LU , Yang S , et al. CircCDKN2B‐AS1 interacts with IMP3 to stabilize hexokinase 2 mRNA and facilitate cervical squamous cell carcinoma aerobic glycolysis progression. J Exp Clin Cancer Res. 2020;39:281. 33308298 10.1186/s13046-020-01793-7 PMC7731507

[32] Samanta S , Guru S , Elaimy AL , et al. IMP3 stabilization of WNT5B mRNA facilitates TAZ activation in breast cancer. Cell Rep. 2018;23:2559‐2567. 29847788 10.1016/j.celrep.2018.04.113 PMC6007887

[33] Bhargava S , Patil V , Shah RA , Somasundaram K . IGF2 mRNA binding protein 3 (IMP3) mediated regulation of transcriptome and translatome in glioma cells. Cancer Biol Ther. 2018;19:42‐52. 28485999 10.1080/15384047.2017.1323601 PMC5790340

[34] Sun C , Zheng X , Sun Y , et al. Identification of IGF2BP3 as an adverse prognostic biomarker of gliomas. Front Genet. 2021;12:743738. 34721530 10.3389/fgene.2021.743738 PMC8551830

[35] Gao S , Gu Y , Niu S , et al. DMDRMR‐mediated regulation of m6A‐modified CDK4 by m6A reader IGF2BP3 drives ccRCC progression. Cancer Res. 2020;81(4):923‐934. 33293428 10.1158/0008-5472.CAN-20-1619

[36] Jia C , Tang H , Yang Y , et al. Ubiquitination of IGF2BP3 by E3 ligase MKRN2 regulates the proliferation and migration of human neuroblastoma SHSY5Y cells. Biochem Biophys Res Commun. 2020;529:43‐50. 32560817 10.1016/j.bbrc.2020.05.112

[37] Jian D , Wang W , Zhou X , et al. Interferon‐induced protein 35 inhibits endothelial cell proliferation, migration and re‐endothelialization of injured arteries by inhibiting the nuclear factor‐kappa B pathway. Acta Physiol. 2018;223:e13037. 10.1111/apha.13037 29350881

[38] Wu B , Mottola G , Schaller M , Upchurch GR Jr , Conte MS . Resolution of vascular injury: specialized lipid mediators and their evolving therapeutic implications. Mol Aspects Med. 2017;58:72‐82. 28765077 10.1016/j.mam.2017.07.005 PMC5660644

[39] Cornelissen A , Vogt FJ . The effects of stenting on coronary endothelium from a molecular biological view: time for improvement? J Cell Mol Med. 2019;23:39‐46. 30353645 10.1111/jcmm.13936 PMC6307786

[40] Pellet‐Many C , Mehta V , Fields L , et al. Neuropilins 1 and 2 mediate neointimal hyperplasia and re‐endothelialization following arterial injury. Cardiovasc Res. 2015;108:288‐298. 26410366 10.1093/cvr/cvv229 PMC4614691

[41] Righi A , Zhang S , Jin L , et al. Analysis of IMP3 expression in normal and neoplastic human pituitary tissues. Endocr Pathol. 2010;21:25‐31. 19898970 10.1007/s12022-009-9096-9

[42] Gao Y , Yang M , Jiang Z , et al. IMP3 expression is associated with poor outcome and epigenetic deregulation in intrahepatic cholangiocarcinoma. Hum Pathol. 2014;45:1184‐1191. 24745619 10.1016/j.humpath.2014.01.016

[43] Liu J , Liu Y , Gong W , et al. Prognostic value of insulin‐like growth factor 2 mRNA‐binding protein 3 and vascular endothelial growth factor‐A in patients with primary non‐small‐cell lung cancer. Oncol Lett. 2019;18:4744‐4752. 31611984 10.3892/ol.2019.10835 PMC6781568

[44] Hammer NA , Hansen T , Byskov AG , et al. Expression of IGF‐II mRNA‐binding proteins (IMPs) in gonads and testicular cancer. Reproduction. 2005;130:203‐212. 16049158 10.1530/rep.1.00664

[45] Yaniv K , Fainsod A , Kalcheim C , Yisraeli JK . The RNA‐binding protein Vg1 RBP is required for cell migration during early neural development. Development. 2003;130:5649‐5661. 14522877 10.1242/dev.00810

[46] Hyun J , Oh SH , Premont RT , Guy CD , Berg CL , Diehl AM . Dysregulated activation of fetal liver programme in acute liver failure. Gut. 2019;68:1076‐1087. 30670575 10.1136/gutjnl-2018-317603 PMC6580749

[47] Yang M , Chen Q , Mei L , et al. Neutrophil elastase promotes neointimal hyperplasia by targeting toll‐like receptor 4 (TLR4)‐NF‐kappaB signalling. Br J Pharmacol. 2021;178:4048‐4068. 34076894 10.1111/bph.15583

[48] Yang Z , Wang T , Wu D , Min Z , Tan J , Yu B . RNA N6‐methyladenosine reader IGF2BP3 regulates cell cycle and angiogenesis in colon cancer. J Exp Clin Cancer Res. 2020;39:203. 32993738 10.1186/s13046-020-01714-8 PMC7523351

[49] Daniel J‐M , Penzkofer D , Teske R , et al. Inhibition of miR‐92a improves re‐endothelialization and prevents neointima formation following vascular injury. Cardiovasc Res. 2014;103:564‐572. 25020912 10.1093/cvr/cvu162 PMC4145012

[50] Dutzmann J , Koch A , Weisheit S , et al. Sonic hedgehog‐dependent activation of adventitial fibroblasts promotes neointima formation. Cardiovasc Res. 2017;113:1653‐1663. 29088375 10.1093/cvr/cvx158

[51] Zhang M , Gao J , Zhao X , et al. p38alpha in macrophages aggravates arterial endothelium injury by releasing IL‐6 through phosphorylating megakaryocytic leukemia 1. Redox Biol. 2021;38:101775. 33171330 10.1016/j.redox.2020.101775 PMC7658717

[52] Wu W , Wang C , Zang H , et al. Mature vascular smooth muscle cells, but not endothelial cells, serve as the major cellular source of intimal hyperplasia in vein grafts. Arterioscler Thromb Vasc Biol. 2020;40:1870‐1890. 32493169 10.1161/ATVBAHA.120.314465 PMC7439253

[53] Wang CH , Lee MF , Yang NI , Mei HF , Lin SY , Cherng WC . Bone marrow rejuvenation accelerates re‐endothelialization and attenuates intimal hyperplasia after vascular injury in aging mice. Circ J. 2013;77:3045‐3053. 24042255 10.1253/circj.cj-13-0267

[54] Peng N , Gao S , Guo X , et al. Silencing of VEGF inhibits human osteosarcoma angiogenesis and promotes cell apoptosis via VEGF/PI3K/AKT signaling pathway. Am J Transl Res. 2016;8:1005‐1015. 27158386 PMC4846943

[55] Suvasini R , Shruti B , Thota B , et al. Insulin growth factor‐2 binding protein 3 (IGF2BP3) is a glioblastoma‐specific marker that activates phosphatidylinositol 3‐kinase/mitogen‐activated protein kinase (PI3K/MAPK) pathways by modulating IGF‐2. J Biol Chem. 2011;286:25882‐25890. 21613208 10.1074/jbc.M110.178012 PMC3138258

[56] Gharib TG , Chen G , Huang CC , et al. Genomic and proteomic analyses of vascular endothelial growth factor and insulin‐like growth factor‐binding protein 3 in lung adenocarcinomas. Clin Lung Cancer. 2004;5:307‐312. 15086970 10.3816/CLC.2004.n.011

[57] Kong X , Xu L , Cao X . Correlations of expressions of IMP3 and VEGF with stage of osteosarcoma, microvascular density and pulmonary metastasis. J BUON. 2020;25:2438‐2443. 33277867

[58] Biswas J , Patel VL , Bhaskar V , Chao JA , Singer RH , Eliscovich C . The structural basis for RNA selectivity by the IMP family of RNA‐binding proteins. Nat Commun. 2019;10:4440. 31570709 10.1038/s41467-019-12193-7 PMC6768852

[59] Nielsen FC , Nielsen J , Christiansen J . A family of IGF‐II mRNA binding proteins (IMP) involved in RNA trafficking. Scand J Clin Lab Invest Suppl. 2001;234:93‐99. 11713986

[60] Findeis‐Hosey JJ , Xu H . Insulin‐like growth factor II‐messenger RNA‐binding protein‐3 and lung cancer. Biotech Histochem. 2012;87:24‐29. 21838610 10.3109/10520295.2011.591831

