# Supplementary figures and images for: IMP3 promotes re‐endothelialization after arterial injury via increasing stability of VEGF mRNAhv
Source: J Cell Mol Med. 2022 Mar 22;26(7):2023–37. doi: 10.1111/jcmm.17225 (PMC8980943; doi:10.1111/jcmm.17225)

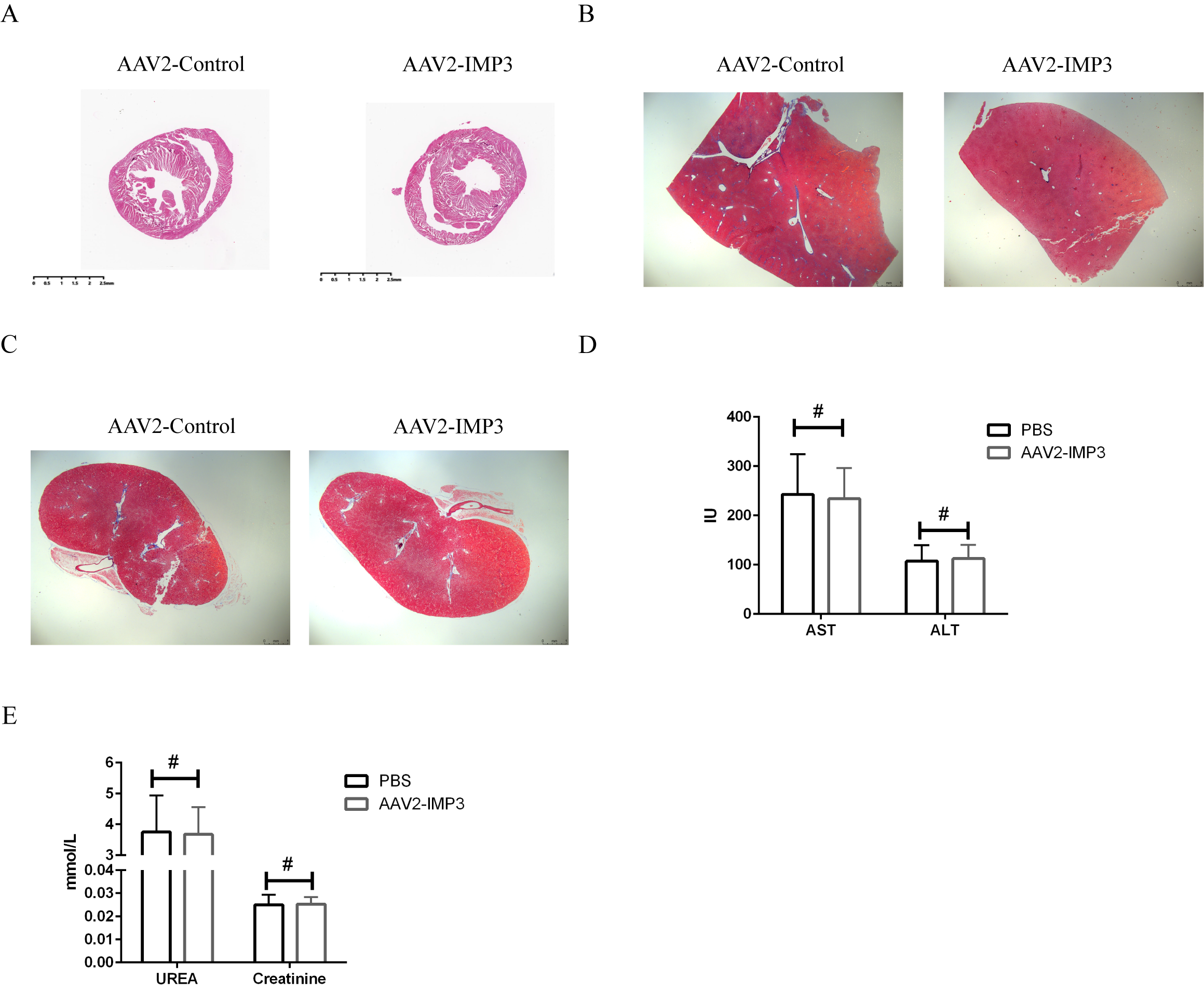

Supplement: Supplementary file 1 — Figure S1 [file JCMM-26-2023-s001.tif]
